# Evaluating the Expression of NOX2 and NOX4 Signaling Pathways in Rats’ Lung Tissues Following Local Chest Irradiation; Modulatory Effect of Melatonin

**DOI:** 10.22088/IJMCM.BUMS.7.4.220

**Published:** 2018-11-21

**Authors:** Masoud Najafi, Alireza Shirazi, Elahe Motevaseli, Ghazale Geraily, Peyman Amini, Dheyauldeen Shabeeb, Ahmed Eleojo Musa

**Affiliations:** 1 *Department of Medical Physics and Biomedical Engineering, Faculty of Medicine, Tehran University of Medical Sciences, Tehran, Iran.*; 2 *Department of Molecular Medicine, School of Advanced Technologies in Medicine, Tehran University of Medical Sciences, Tehran, Iran.*; 3 *Department of Radiology, Faculty of Paramedical, Tehran University of Medical Sciences, Tehran, Iran.*; 4 *Department of Medical Physics and Biomedical Engineering, Faculty of Medicine, Tehran University of Medical Sciences (International Campus), Tehran, Iran.*; 5 *Department of Physiology, College of Medicine, University of Misan, Misan, Iraq.*; 6 *Research Center for Molecular and Cellular Imaging, Tehran University of Medical Sciences (International Campus), Tehran, Iran.*

**Keywords:** Radiation, lung, NOX2, NOX4, TGFβR1, SMAD2

## Abstract

Lung injury is one of the major concerns for chest cancer patients that undergo radiotherapy as well as persons exposed to an accidental radiological event. Reduction/oxidation (redox) system plays a key role in lung injury via chronic upregulation of pro-oxidant enzymes. *NOX2* and *NOX4 *are two important reactive oxygen species generating enzymes that are involved in radiation toxicity in some organs such as the bone marrow. In this study, we aimed to evaluate the expression of *NOX2* and *NOX4* signaling in rat’s lung tissues. Upregulation of these genes may be involved in radiation-induced lung injury. Moreover, we evaluated the role of pre-treatment with melatonin on the expression of these genes. Twenty male rats were divided into 4 groups as control; melatonin treated; irradiation; and irradiation with melatonin pre-treatment. Rats were exposed to 15 Gy ^60^Co gamma rays and sacrificed after 10 weeks for evaluation of *NF-κB*, *TGFβR1*, *SMAD2*, *NOX2*, and *NOX4* gene expression by real-time PCR. Results showed the upregulation of all five genes. The expression of *NOX2* was more obvious compared to other genes. Administration of melatonin before irradiation could attenuate the expression of all mentioned genes. Results indicate that upregulation of NADPH oxidase genes such as *NOX2* and *NOX4* may be involved in the late effects of lung exposure to ionizing radiation. Melatonin via downregulation of these pro-oxidant genes is able to attenuate radiation toxicity in the lung.

The lung is one of the most radiosensitive organs, and also a late responding one. Radiation therapy is a common treatment option for patients with lung, head and neck or breast cancers (1). For patients who receive radiation to the chest area such as total body irradiation (TBI), lung damage is one of the most common side effects (2). Moreover, pneumonitis and fibrosis are two main limiting factors (3). In addition to clinical importance of these side effects, fibrosis and pneumonitis pose a threat to the lives of people exposed to accidental nuclear or radiological events (4). Lung reactions to ionizing radiation may appear after several months to years following exposure to radiation. A major symptom of lung injury is difficulty in breathing, resulting from massive collagen deposition which could lead to loss of lung function (5).

Treatment or prevention of radiation-induced lung pneumonitis and fibrosis requires a knowledge about the mechanisms involved in these processes. Studies have revealed that increased secretion of some cytokines and growth factors such as interleukin (IL)-1, IL-4, IL-6, IL-13, transforming growth factor beta (TGF-β), platelet-derived growth factor (PDGF), tumor necrosis factor alpha (TNF-α) as well as immune mediators such as signal transducer and activator of transcription (STAT) family, cyclooxygenase-2 (COX-2), mothers against decapentaplegic homolog 2 (known as SMAD2), and mitogen- activated protein kinases (MAPKs) are involved in the late effects of exposure to ionizing radiation in several organs, including the lung (6-8). These cytokines and growth factors stimulate the production of free radicals and matrix metalloproteinase enzymes that lead to infiltration of inflammatory cells, accumulation of collagen, and differentiation of myofibroblasts (9). Studies have proposed that stimulation of reduction/oxidation (redox) reactions by these cytokines and immune mediators have a key role in chronic oxidative damage and development of late effects of ionizing radiation (10). Studies have shown that overproduction of reactive oxygen species (ROS) by mitochondria as well as upregulation of NADPH oxidase genes play a central role in radiation toxicity (11). Amongst the different types of NADPH oxidase subfamilies, *NOX1*, *NOX2*, *NOX4* and dual oxidases including *DUOX1* and *DUOX2* have shown to be involved in radiation-induced chronic oxidative stress in some organs. Suppression of these genes has been proposed as a strategy for the amelioration of side effects of radiation in mice bone marrow (10).

So far, no complete treatment has been proposed for patients suffering from radiation-induced lung injury. However, in experimental studies, various types of radiation modifiers such as natural antioxidants, herbal agents or some man-made drugs have been proposed for this aim (12-14). Melatonin, a natural human body agent has shown potent radioprotective effect against detrimental toxic consequences of ionizing radiation. Some studies have proposed that the protective effect of melatonin may be mediated though modifications in the expression of some genes involved in inflammatory and redox reactions (15, 16). In the present study, we assessed the expression of *TGFβR1*, *SMAD2*, nuclear factor-κB (*NF-κB*), *NOX2*, and *NOX4*. NOX2 is a membrane protein that can be stimulated by TGFβR1. In addition, TGFβR1 through some mediators such as SMAD2 and NF-κB can upregulate *NOX4* gene expression. We also evaluated the effect of pre-treatment with melatonin on the expression of these signaling pathways.

## Materials and methods

**Experimental design**

In this experimental study, 20 male Wistar rats (200 ± 20 g) were used. All rats were purchased from the Razi Institute, Tehran University of Medical Sciences, Tehran, Iran. This experimental study was in accordance with the “Guide for the care and use of laboratory animals” of Tehran University of Medical Sciences. The rats were kept under suitable conditions of temperature (23 ± 2 ^o^C), humidity (55%) as well as light and dark cycle of 8:00 to 20:00 and 20:00 to 8:00, respectively. The rats were divided into 4 groups as group 1: control without any contravention except anesthesia with ketamine and xylazine similar to other groups; group 2: melatonin treated with 100 mg/kg; group 3: irradiation only; group 4: melatonin treated before irradiation. After 10 weeks, all rats were sacrificed and their lung tissues were extracted, then frozen at -80 ^o^C.

**Irradiation and drug treatment**

Melatonin (Sigma-Aldrich, USA) was dissolved in 20% ethanol at a concentration of 20 mg/ml. 1 ml melatonin solution (equal to 100 mg/kg) was injected intraperitoneally 30 minutes before irradiation. All rats were anesthetized using an appropriate dose of ketamine and xylazine mixture prior to irradiation. Irradiation was performed using a cobalt-60 (^60^Co) gamma ray source. Rats in groups 3 and 4 were irradiated with 15 Gy ^60^Co (1.25 MeV) at a dose rate of 109 cGy/min and SSD of 60 cm.

**RNA isolation, cDNA synthesis, and Real Time PCR**

For the quantification of gene expressions, real-time PCR (RT-PCR) was carried out on rats’ lung samples from all groups. At first, lung tissues were homogenized and total RNA was extracted using an RNX kit (Sinaclon, Iran) according to the manufacturer's instruction. Afterwards, cDNA was produced with total RNA using the cDNA synthesis kit (GeneAll, South Korea). The reverse transcription products were used for real-time PCR. PCR reactions were performed in a volume of 10 µl containing 5 pmole/µl each forward and reverse primers, 5 µl SYBR Green master mix (Takara, Japan), and 4 µl distilled water. The primer sequences of genes, including *NF-κB*, *TGFβR1*, *SMAD2*, *NOX2*, and *NOX4* are shown in Table 1. *GAPDH* was selected as internal control. Primers were first evaluated in Gene Runner software and then blasted in NCBI.

All samples were run in duplicate. In each sample, ΔCT was calculated using differences between the mean CT of target or housekeeping genes. Then, ΔΔCT was calculated using differences between the mean ΔCT for treatment groups and mean ΔCT for control group. Finally, the relative fold changes in the expression of our target genes including *TGFβR1*, *NF-κB*, *SMAD2*, *NOX2*, and *NOX4* were calculated compared to the internal housekeeping gene.

**Statistical analyses**

All statistical analyses were performed using Statistical Package for Social Sciences (SPSS) software (version 16, SPSS, Inc, Chicago, IL, USA). The significance of reported findings was conducted with T-Test. P values < 0.05 were considered to be statistically significant.

**Table 1:** The primer sequences for real-time PCR

***Genes ***	**Forward sequence**	**Reverse sequence **
***TGF*** ***β*** ***R1***	TGCACCATCTTCAAAAACAGGG	CAGCTGACTGCTTTTCTGTAGT
***NF-κB***	AATTGCCCCGGCAT	TCCCGTAACCGCGTA
***SMAD2***	TCTCCGGCTGAACTGTCTCCTA	GCGATTGAACACCAAAATGCA
***NOX2***	CTGCCAGTGTGTCGGAATCT	TGTGAATGGCCGTGTGAAGT
***NOX4***	GGATCACAGAAGGTCCCTAGC	AGAAGTTCAGGGCGTTCACC
***GAPDH***	AGTGCCAGCCTCGTCTCATA	ATGAAGGGGTCGTTGATGGC

**Fig. 1:**
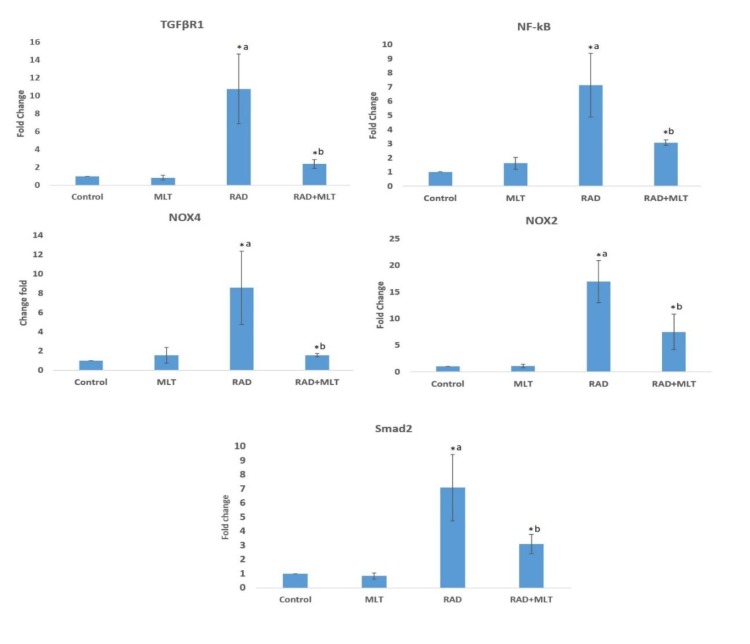
Effects of melatonin treatment before chest irradiation on genes expression levels. Changes in the expression of TGFBR1, NF-κB, SMAD2, NOX2, and NOX4 following melatonin treatment, irradiation, and melatonin treatment before irradiation. RAD: radiation; MLT: melatonin; a: significant compared to control group; b: significant compared to radiation group (t-test, P < 0.05)

## Results

Real-time PCR results showed that irradiation of rat’s lung with 15 Gy gamma rays led to a significant increase in the expression of *TGFβR1* when compared with the control group (10 ± 3.9) (P =0.008). Treatment with melatonin before irradiation led to a significant attenuation of *TGFβR1* expression (2.38 ± 0.50) in comparison with the irradiation group (P = 0.012). Treatment with melatonin alone did not cause any significant change in the basal level of *TGFβR1 *expression (0.84 ± 25). The results of *NF-κB* expression showed that irradiation caused a significant increase in its expression in comparison with the control group (7.14 ± 02.25) (P = 0.007). Administration of melatonin before irradiation could attenuate the expression of *NF-κB *in comparison with the irradiation group (3.06 ± 0.18) (P = 0.03). Furthermore, treatment with melatonin alone did not cause any change in the expression of *NF-κB* (1.6 ± 042).

Real-time PCR results showed that in response to lung irradiation, the expression of *NOX2* was upregulated. The expression of this gene increased by 16.96 ± 3.96 fold in comparison with the control group (P = 0.001). When rats were treated with melatonin before exposure to gamma rays, the expression of *NOX2* was attenuated significantly (7.50 ± 3.30) (P = 0.008). Similar to *NOX2*, irradiation also caused the upregulation of *NOX4*. Results showed that irradiation of rats’ lungs led to 8.55 ± 3.80 fold increase in the expression of *NOX4* (P = 0.006) while melatonin administration before exposure to gamma rays caused significant decrease in its expression in comparison with the radiation group (1.56 ± 16) (P = 0.01). Similar to other genes, the expression of *SMAD2* was upregulated following irradiation of rats’ lungs (7.09 ± 2.33 fold) (P = 0.032). Treatment with melatonin before irradiation led to a significant attenuation of *SMAD2* expression (3.10 ± 0.68) (P =0.002). The expression level variations of all studied genes are represented in figure 1.

## Discussion

Lung toxicity is one of the main limiting factors for radiotherapy or radio/chemotherapy of patients with chest cancer. Although, lung reactions are not observed at the early phase of radiotherapy, pneumonitis or fibrosis may cause severe reactions or death in patients (17). This has been observed in patients with other cancers such as thyroid cancer with metastasis in the lung (18). Moreover, late effects of lung exposure to high doses of ionizing radiation is a threat to people after a radiological or nuclear event. Chronic inflammation and oxidative stress can be involved in carcinogenesis and death by pneumonitis and fibrosis in exposed people (19). As emerging evidences show that chronic upregulation of pro-inflammatory and pro-fibrotic cytokines are involved in the late effects of lung exposure to ionizing radiation, knowledge of molecular mechanisms involved in these processes can help alleviate them. TGFβR1 is the main receptor of TGF-β which plays a key role in the development of radiation-induced chronic oxidative stress and fibrosis. Suppression of this cytokine or its receptor has been proposed for the mitigation of radiation-induced lung injury (20).

In the present study, we aimed to evaluate the expression of two important pro-oxidant enzymes: NOX2 and NOX4 that may be involved in late effects of exposure to ionizing radiation. Our study showed a high expression of *NOX2* and *NOX4* following irradiation of rats’ lung. In addition, our results showed increased levels of upstream genes like *TGFβR1*, *SMAD2* and *NF-κB*. However melatonin treatment alone did not affect the basal expression level of these genes, when administered before irradiation it could attenuate the upregulation of these genes. Melatonin could potently suppress *TGFβR1* and *NOX4* expression, while in comparison to these genes, it has lower effect on *NOX2* and *SMAD2*.

Previous studies have suggested that TGF-β plays a key role in the stimulation of *NOX2* and *NOX4* gene expression. It was shown that TGF-β targeting was associated with downregulation of *NOX2* and *NOX4*, decreased ROS level as well as attenuation of bone marrow injury following whole body irradiation of mice (21). Direct targeting of *NOX4* has shown similar results (22). In recent years, studies have shown that chronic ROS/NO production plays a key role in both acute and late effects of radiation. Studies indicated that these enzymes via continuous production of free radical amplify disruption of normal function of irradiated organs (23). Melatonin, via attenuation of the expression of these genes is able to mitigate radiation injury in lung tissue.

The present study showed that exposure to a high dose of ionizing radiation can induce chronic upregulation of NADPH oxidase genes including *NOX2* and *NOX4*. It is possible that continuous upregulation of these genes may be involved in late effects of lung exposure to ionizing radiation. Chronic upregulation of these genes may induce further redox reactions and amplify inflammation and fibrosis in the lung. Melatonin has been proposed as a potent anti- inflammatory and anti-fibrotic agent. This study reports that attenuation of *NOX2* and *NOX4* by melatonin may be one of the mechanisms of its radioprotective effect.
